# Advances in mRNA Vaccines for Infectious Diseases

**DOI:** 10.3389/fimmu.2019.00594

**Published:** 2019-03-27

**Authors:** Cuiling Zhang, Giulietta Maruggi, Hu Shan, Junwei Li

**Affiliations:** ^1^College of Veterinary Medicine, Qingdao Agricultural University, Qingdao, China; ^2^GSK, Rockville, MD, United States

**Keywords:** mRNA vaccine, infectious disease, delivery, mechanism, application

## Abstract

During the last two decades, there has been broad interest in RNA-based technologies for the development of prophylactic and therapeutic vaccines. Preclinical and clinical trials have shown that mRNA vaccines provide a safe and long-lasting immune response in animal models and humans. In this review, we summarize current research progress on mRNA vaccines, which have the potential to be quick-manufactured and to become powerful tools against infectious disease and we highlight the bright future of their design and applications.

## Introduction

Vaccination is the most successful medical approach to disease prevention and control. The successful development and use of vaccines has saved thousands of lives and large amounts of money. In the future, vaccines have the potential to be used not only against infectious diseases but also for cancer as a prophylactic and treatment tool, and for elimination of allergens (1–3). Prior to the 1980s, vaccines were developed for protection against disease-causing microorganisms. Empirically, inactivated vaccines were produced by heat or chemical treatment, and live attenuated vaccines were generally developed in animals, cell lines or unfavorable growth conditions. During vaccine development, the mechanisms involved in conferring immunity were unknown. Nevertheless, the use of live attenuated or killed whole organism-based vaccines had enormous success in the control and eradication of a number of severe human infectious diseases, including smallpox, polio, measles, mumps, rubella, and animal infectious disease, such as classic swine fever, cattle plague, and equine infectious anemia. More recently, live attenuated (LAV), subunit and peptide based vaccines have been developed thanks to advancements in molecular biology theory and technologies. The results obtained with LAV vaccination dramatically expanded our knowledge of the mechanisms related to the immune response elicited by these vaccines. For inactivated vaccines, antigen-specific antibodies largely contribute to the prevention and control of microbe-initiated infectious disease. In addition to specific humoral immune responses. LAVs elicit strong cellular immune responses, which are critical to eradicate many intracellular pathogens. Nevertheless, the failures that are sometimes caused by inactivated vaccines are ascribed to mutation of the surface antigens of pathogens. Additional concerns about LAV applications include the potential to cause disease in immuno-compromised individuals and the possibility of reversion to a virulent form due to the back-mutation, the acquisition of compensatory mutations, or recombination with circulating transmissible wild-type strains (4, 5). Nevertheless, subunit and peptide vaccines are less effective at eliciting a robust CD8^+^ immune response, which is important for intracellular pathogens, including viruses and some bacteria (6, 7).

**Figure 1:**
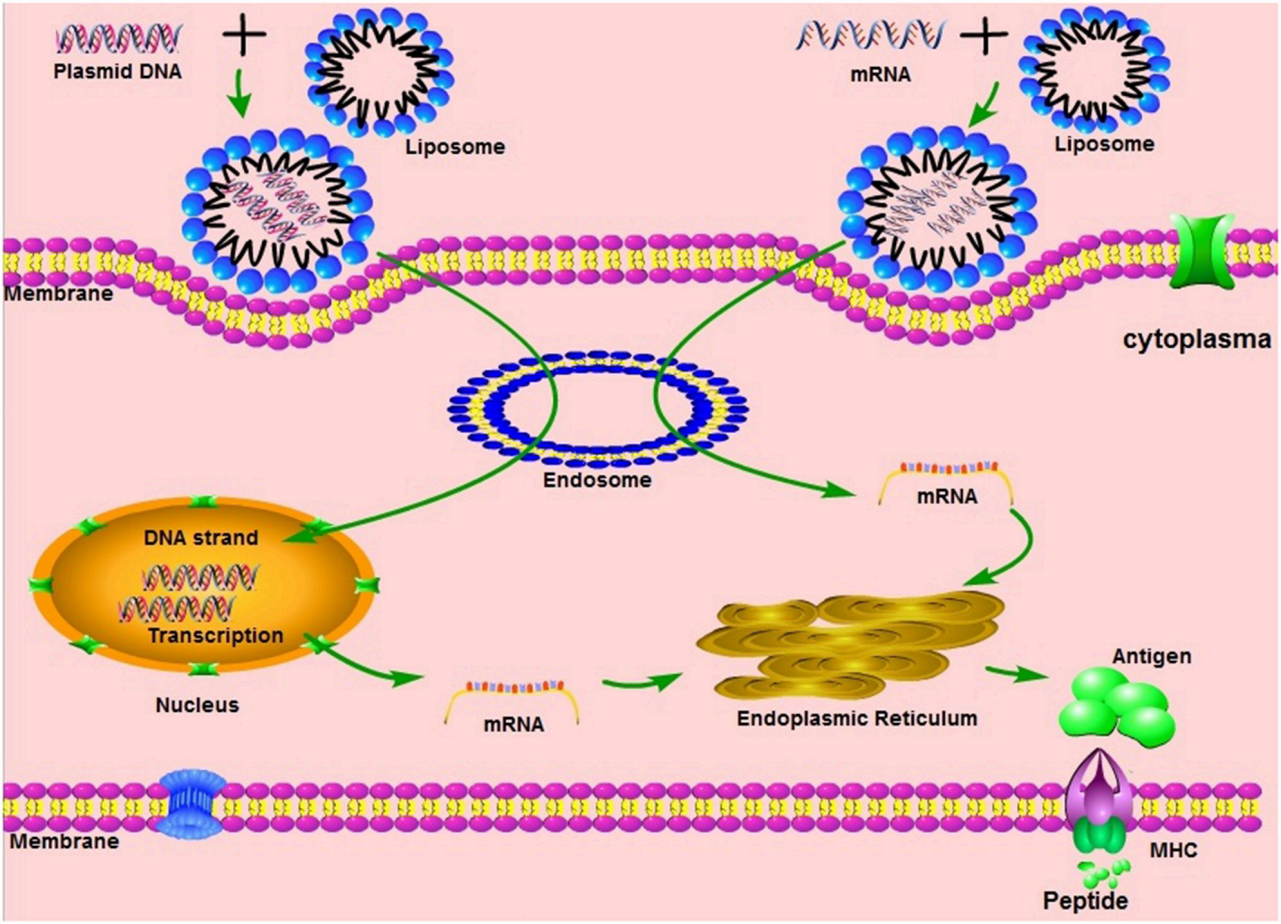
The mechanisms of different nucleic acid vaccines, including DNA vaccines, mRNA vaccines. MHC, Major histocompatibility complex.

Vaccination with non-viral delivered nucleic acid-based vaccines mimics infection or immunization with live microorganisms and stimulates potent T follicular helper and germinal center B cell immune response (8, 9). Furthermore, non-viral delivered nucleic acid-based vaccine manufacturing is safe and time-saving, without the growth of highly pathogenic organisms at a large scale and less risks from contamination with live infectious reagents and the release of dangerous pathogens. Notably, for most emerging and re-emerging devastating infectious diseases, the main obstacle is obtaining a stockpile in a short timeframe (10). Non-viral delivered nucleic acid-based vaccines can fill the gap between a disease epidemic and a desperately needed vaccine (10). Non-viral delivered nucleic acids are categorized as DNA or RNA according to their type of 5-carbon sugar. From being administrated to antigen expression, DNA vaccine and RNA vaccines are processed through different pathways. In the steps between immunization with a DNA template and expression of the target antigen, the DNA has to overcome the cytoplasmic membrane and nuclear membrane, be transcribed into mRNA, and move back into the cytoplasm and initiate translation (refer to Figure 1). Although promising and with shown safety, well-tolerability and immunogenicity, DNA vaccines were characterized by suboptimal potency in early clinical trials (11). Enhanced delivery technologies, such as electroporation, have increased the efficacy of DNA vaccines in humans (12), but have not reduced the potential risk of integration of exogenous DNA into the host genome, which may cause severe mutagenesis and induced new diseases (13, 14). Since naked *in vitro* transcribed mRNA was found to be expressed *in vivo* after direct injection into mouse muscle, mRNA has been investigated extensively as a preventive and therapeutic platform (15–19). Due to the dramatic development of RNA-based vaccine studies and applications, a plethora of mRNA vaccines have entered into clinical trial (19). Comparatively, mRNA vaccines confer several advantages over viral vectored vaccines and DNA vaccines (summary in Table 1). The utilization of RNA as a therapeutic tool is not the focus of this manuscript and has been extensively reviewed elsewhere (2, 19, 20). In this review, we provide highlights on mRNA vaccines as promising tools in the prevention and control of infectious disease.

**Table 1:** Advantages and disadvantages of viral vectored vaccines, DNA vaccines and RNA vaccines.

**Vaccines**	**Advantages**	**Disadvantages**
Viral vectored vaccines	Stimulation of innate immune response; induction of T and B cell immune response.	induction of anti-vector immunity: cell based manufacturing
DNA vaccines	Non-infectious; stimulation of innate immune response; egg and cell free; stable, rapid and scalable production; induction of T and B cell immune response.	Potential integration into human genome; poor immunogenicity in humans.
RNA vaccines	Non-infectious, non-integrating, natural degradation, egg and cell free, rapid and scalable production; stimulation of innate immune response; induction of T and B cell immune response.	Concerns with instability and low immunogenicity.

## Conception and Forms of mRNA Vaccines

mRNA vaccines were reported to be effective for direct gene transfer for the first time by Woff et al. (15). Currently, two forms of mRNA vaccines have been developed: conventional mRNA vaccines and self-amplifying mRNA vaccines, which are derived from positive strand RNA viruses. Although mRNA vaccines were first tested in the early 1990s, these vaccines were not initially extensively utilized due to concerns about their fragile stability caused by omnipresent ribonucleases and small-scale production. Initial demonstration that mRNA stability can be improved by optimization and formulation was published by Ross and colleagues in 1995 (21). Since that time, studies on mRNA vaccines have exploded and mRNA can now be synthetically produced, through a cell-free enzymatic transcription reaction. The *in vitro* transcription reaction includes a linearized plasmid DNA encoding the mRNA vaccine, as a template, a recombinant RNA polymerase, and nucleoside triphosphates as essential components. A cap structure is enzymatically added to the transcriptional product at the end of the reaction or as a synthetic cap analog in a single step procedure. Finally, a poly(A) tail will be provided to form a mature mRNA sequence.

Conventional mRNA vaccines include in their simplest an ORF for the target antigen, flanked by untranslated regions (UTRs) and with a terminal poly(A) tail. After transfection, they drive transient antigen expression. In addition to conventional vaccines, there is another mRNA vaccine platform based on the genome of positive strand viruses, most commonly alphaviruses. These mRNA vaccines are based on an engineered viral genome containing the genes encoding the RNA replication machinery whereas the structural protein sequences are replaced with the gene of interest (GoI) and the resulting genomes are referred as replicons. These vaccines are named self-amplifying mRNA and are capable of directing their self-replication, through synthesis of the RNA-dependent RNA polymerase complex, generating multiple copies of the antigen-encoding mRNA, and express high levels of the heterologous gene when they are introduced into the cytoplasm of host cells, in a way that mimics production of antigens *in vivo* by viral pathogens, triggering both humoral and cellular immune responses (22–27). Self-amplifying mRNA can be derived from the engineered genomes of Sindbis virus, Semliki Forest virus, Kunjin virus, among others (28–30). Self-amplifying mRNAs (~9–11 kb) are generated from the DNA template with similar procedures to those previously described for conventional mRNAs and RNA molecules can be produced at a large scale *in vitro*. After the purified RNA replicon is delivered into host cells, either as viral particles or as synthetically formulated RNA, it is translated extensively and amplified by its encoding RNA-dependent RNA polymerase. Compared with the rapid expression of conventional mRNAs, published results have shown that vaccination with self-amplifying mRNA vaccines results in higher antigen expression levels, although delayed in time, which persist for several days *in vivo*. Equivalent protection is conferred but at a much lower RNA dose (31). Due to the lack of viral structural proteins, the replicon does not produce infectious viral particles. Additionally, both conventional mRNA and self-amplifying mRNAs cannot potentially integrate into the host genome and will be degraded naturally during the process of antigen expression. These characteristics indicate that mRNA vaccines have the potential to be much safer than other vaccines and are a promising vaccine platform.

## Engineered mRNA With Potent Efficiency

Stability and translation of mRNA is crucial for a successful RNA vaccine (32, 33). In the process of translation, mRNA purity is critical to determine its stability and protein yield (34). Contamination with dsRNAs, derived from aberrant RNA polymerase activities, leads to the inhibition of translation and degradation of cellular mRNA and ribosomal RNA, thus decreasing protein expression by interrupting the translation machine. The removal of dsRNA can increase translation dramatically (35). Excess components and short or double strand RNAs (dsRNA) can be removed by purification. Initially, lithium chloride (LiCl) was used for this purpose, but it restricted the industrialization of mRNA vaccines and it did not remove dsRNAs. Purification via fast protein liquid chromatography (FPLC) or high-performance liquid chromatography (HPLC) could be utilized to remove any remaining product and produce mRNA at a large scale and for Good Manufacturing Practice (GMP) processes (35–37). Non-coding sequence flanking 5′ and 3′ terminal of open reading frame (ORF) is crucial for translation. The 5′ untranslated region, such as kozak sequence, or 5′ caps is required for efficient protein production (38–40). The 3′ untranslated region containing optimal poly(A) signal determined the stability of mRNA and increased protein translation (41–45). Additionally, codon optimization is a popular method to avoid rare codons with low utilization, to increase protein production, mRNA abundance and stability (46–49).

mRNA vaccines are efficient at antigen expression, but sequence and secondary structures formed by mRNAs are recognized by a number of innate immune receptors, and this recognition can inhibit protein translation. Thanks to advancement in RNA biology understanding, several methods can be employed to increase the potency of mRNA vaccines, including sequence optimization and usage of modified nucleosides. Recognition from innate immune sensors can be avoided by incorporating modified nucleosides, such as pseudouridine (Ψ), 5-methylcytidine (5 mC), cap-1 structure and optimized codons, which in turns improve translation efficiency (50–55). During the *in vitro* transcription of mRNA, immature mRNA would be produced as contamination which inhibited translation through stimulating innate immune activation. FPLC and HPLC purification could tackle this problem (35, 37).

Currently, most vaccines in use, with the exception of some animal vaccines, need to be transported and stored in an uninterrupted cold-chain process, which is prone to failure, especially in poor rural areas of tropical countries; these requirements are not being met by available effective vaccines to prevent and control infectious diseases. Therefore, the development of thermostable vaccines has been gaining interest. Optimization in formulation of synthetic mRNA vaccines have shown that it is possible to generate thermostable vaccines. The results described by Jones showed that freeze-dried mRNA with trehalose or naked mRNA is stable for at least 10 months at 4°C. After being transfected, these mRNAs expressed high levels of proteins and conferred highly effective and long-lasting immunity in newborn and elderly animal models (56). Another lyophilized mRNA vaccine was shown to be stable at 5–25°C for 36 months and 40°C for 6 months (57). Stitz and colleagues showed that when a protamine-encapsulated conventional mRNA-based rabies virus vaccine was subjected to oscillating temperatures between 4 and 56°C for 20 cycles and exposure 70°C, its immunogenicity and protective effects were not compromised (58). Encapsulation of mRNA with cationic liposome or cell penetrating peptide (CPP) protected mRNA from degradation by RNase. These intriguing approaches would be discussed in delivery methods.

## RNA Vaccines in the Prevention of Infectious Disease

During the last two decades, mRNA vaccines have been investigated extensively for infectious disease prevention, and for cancer prophylaxis and therapy. Much progress has been made thus far (19, 20). Cancer mRNA vaccines were designed to express tumor-associated antigens that stimulate cell-mediated immune responses to clear or inhibit cancer cells (59). Most cancer vaccine are investigated more as therapeutics than prophylactics and have been reviewed elsewhere (20, 60, 61). mRNA vaccines against infectious diseases could be developed as prophylactic or therapeutic. mRNA vaccines expressing antigen of infectious pathogen induce both strong and potent T cell and humoral immune responses (8, 16, 19). As previously described the production procedure to generate mRNA vaccines is entirely cell-free, simple and rapid if compared to production of whole microbe, live attenuated and subunit vaccines. This fast and simple manufacturing process makes mRNA a promising bio-product that can potentially fill the gap between emerging infectious disease and the desperate need for effective vaccines. Producing RNA at a large scale to satisfy commercialization is the first step toward making mRNA vaccines. Currently, all components needed for mRNA production are available at the GMP grade; however, some components are supplied at a limited scale.

A great deal of research has been initially conducted on the development of cancer mRNA vaccines and has demonstrated the feasibility of producing clinical grade *in vitro* transcribed RNA (60). Several projects on mRNA vaccines against infectious disease have also been conducted, although clinical evaluation is still limited. For example, several RNA-based vaccine platforms have been utilized for the development of influenza vaccines. Several published results showed that RNA-based influenza vaccines induce a broadly protective immune response against not only homologous but also hetero-subtypic influenza viruses (62–66). Influenza mRNA vaccines hold great promises being an egg-free platform, and leading to production of antigen with high fidelity in mammalian cells. Recent published results demonstrated that the loss of a glycosylation site by a mutation in the hemagglutinin (HA) of the egg-adapted H3N2 vaccine strain resulted in poor neutralization of circulating H3N2 viruses in vaccinated humans and ferrets. In contrast, the process of mRNA vaccine production is egg-free, and mRNA-encoded proteins are properly folded and glycosylated in host cells after vaccine administration, thus avoiding the risk of producing incorrect antigens (67, 68).

mRNA has also been used in the veterinary field to prevent animal infectious diseases. Pulido et al. demonstrated that immunization with *in vitro* transcribed mRNA induced protection against foot and mouse disease virus in mice (69). Saxena and colleagues demonstrated that a self-amplifying mRNA vaccine encoding rabies virus glycoprotein induced an immune response and provided protection in mice and could potentially be used to prevent rabies in canine (70). Recently, VanBlargan et al. developed a lipid nanoparticle (LNP)-encapsulated modified mRNA vaccine encoding prM and E genes of deer powassan virus (POWV). This mRNA vaccine induced robust humoral immune response not only against POWV strains but also against the distantly related Langat virus (71). As described previously, modification of nucleosides and optimization of codons can avoid recognition by innate immune sensors to improve translation efficiency. In Table 2, studies conducted with nucleoside modified and non-modified mRNA vaccines for infectious disease are summarized (52, 58, 72–78).

**Table 2:** Nucleoside modified or non-modified mRNA vaccines against infectious diseases.

**Targets**	**Routes**	**Formulation**	**Immune response**	**Animal models**	**References**
prM-E, Zika virus	i.d.	mRNA-LNP	Humoral	Mice and NHP	Pardi et al. (74)
HA, influenza virus	i.d.	Complex with protamine	Humoral and cellular	Mice, ferrets, and pigs	Petsch et al. (76)
prM-E, Zikavirus	i.m.	LNP	Humoral	Mice	Richner et al. (75)
GP, rabies virus	i.d.	Complex with protamine	Humoral and cellular	Mice and pigs	Schnee et al. (77)
GP, rabies virus	i.d.	Complex with protamine	Humoral	Mice	Stitz et al. (58)
GP, Ebola virus	i.m.	LNP	Humoral	Guinea pigs	Meyer et al. (52)
NP, influenza virus	s.c.	Liposome-entrapped	Humoral and cellular	Mice	Martinon et al. (72)
Gag, HIV	s.c.	Self-assembled cationic nanomicelles	Humoral	Mice	Zhao et al. (73)
Env, HIV	i.d.	LNP	Humoral and cellular	Mice	Pardi et al. (74)
IgG, HIV	i.v.	LNP	Humoral	Humanized mice	Pardi et al. (78)
prM and E POWV	i.m.	LNP	Humoral	mice	VanBlargan et al. (71)

*prM-E, premembrane and envelope; NHP, nonhuman primates; id., intradermal; LNP, lipid nanoparticle; i.m., intramuscular; s.c., subcutaneous; i.v., intravenous; HA, hemagglutinin; POWV, Powassan virus*.

Besides being used as vaccine, mRNA could also be deployed for therapeutic purposes. Interestingly, a recent publication by Pardi and colleagues showed that the adnimistration of mRNA encoding the light and heavy chains of a broadly neutralizing anti-HIV antibody encapsulated in lipid nanoparticles (LNPs) protected humanized mice from intravenous HIV challenge (79). The data suggest that the utilization of nucleoside-modified mRNA can be expanded for passive immunotherapy against HIV, cytomegalovirus (CMV), human papiloma virus, etc. Self-amplifying mRNA vaccines enable large amounts of prompt antigen expression and potent T cellular immune responses. In Table 3, we summarize publications on self-amplifying mRNA vaccines for infectious disease, delivered as viral replicon particles or synthetic formulated mRNA (80, 82–85, 87, 91, 92).

**Table 3:** Self-amplifying mRNA vaccines against infectious diseases.

**Replicons**	**Targets**	**Immune response**	**Animal models**	**References**
N/A	HA, influenza virus	Humoral and cellular	Mice	Brazzoli et al. (65)
N/A	M1, NP, influenza virus	Humoral and cellular	Mice	Magini et al. (63)
VEEV	E85, dengue virus	Humoral and cellular	Mice	Khalil et al. (80)
SFV	NS3, hepatitis C virus	Humoral and cellular	Mice	Lundstrom et al. (81)
KUNV	GP, Ebola virus	Humoral and cellular	NHP	Pyankov et al. (82)
RVFV	HA, influenza virus	Humoral and cellular	Mice	Oreshkova et al. (83)
SFV	E6, E7, papilloma virus	Humoral and cellular	Mice	Van de Wall et al. (84)
TBEV	Capsid protein C, TBEV	Humoral and cellular	Mice	Aberle et al. (92)
N/A	Gag, HIV	Humoral and cellular	NHP	Bogers et al. (85)
KUNV	Gag, HIV	Humoral	Mice	Harvey et al. (86)
JEV	Epitope SP70, EV71	Humoral and cellular	Mice	Huang et al. (87)
VEEV	Pentamer, CMV	Humoral and cellular	Mice	Hofmann et al. (88)
SFV	prM-E, loupingill virus; HA, influenza; F, RSV	Humoral and cellular	Mice	Fleeton et al. (62)
N/A	F, RSV	Humoral and cellular	Mice	Geall et al. (22)
VEEV, SFV	HA, influenza virus; GP, Ebola virus	Humoral and cellular	Mice	Chahal et al. (64)
N/A	SLOdm and BP-2a Streptococci	Humoral	Mice	Maruggi et al. (89)
SFV	Conserved region, HIV	cellular	Mice	Moyo et al. (90)

*VEEV, Venezuelan equine encephalitis virus; HA, hemagglutinin; SFV, Semliki Forest virus; KUNV, Kunjinvirus; RVFV, Rift Valley fever virus; JEV, Japanese encephalitis virus; NHP, nonhuman primate; TBEV, tick-borne encephalitis virus; CMV, cytomegalovirus; HIV, human immunodeficiency virus; SLOdm, GAS streptolysin-O; BP-2a, GBS pilus 2a backbone protein; N/A, not known*.

## Delivery Route and Formulation of mRNA Vaccines

The administration route and formulation of mRNA vaccines are crucial to determine the kinetics and magnitude of antigen expression as well as the potency of the immune response. For example, intravenous administration of unmodified naked mRNA resulted in rapid digestion by ribonucleases and stimulation of the innate immune response, but these limitations can be overcome by appropriate delivery systems and mRNA modifications (93). mRNA vaccines are administered via a systemic or local method based on antigen expression localization requirements. Direct intramuscular (i.m), intradermal (i.d.) or subcutaneous injection of *in vitro* transcribed mRNA are the main delivery routes for mRNA vaccines against infectious diseases, while intraperitoneal (i.p.) and intravenous (i.v.) administration are employed when systemic expression of antigens of interest is needed, mostly for therapeutic applications. Multiple reports have been recently published and showed that a variety of antigens can be expressed with high efficiency and induced potent humoral and cellular immune responses after mRNA vaccination. Lipid nanoparticles (LNP) loaded with nucleoside modified conventional mRNA encoding firefly luciferase have been used, for example, to examine the influence of route of administration on kinetics of antigen expression (94). i.m. and i.d. injections offered the best levels and duration of effect, with protein production peaking at 4 h and maintained locally 8–10 days post injection, depending on the dose. Both i.m. and i.d. administration in Rhesus macaques with nucleoside modified conventional mRNA encoding influenza H10 encapsulated in LNP induced protective titers, but this response occurred more rapidly by i.d. administration than by i.m. administration (95).

CV7201 is an mRNA vaccine candidate under development by CureVac AG. i.d. and i.m. injection ofCV7201 in mice and pigs induced potent humoral and T cell immune responses (77). In a phase I clinical trial, CV7201 showed long-term safety and immunogenicity against the rabies virus at alow dose. No differences were observed in terms of safety between i.d and i.m. administration or between needle-syringe or needle-free injection of CV7201. However, when neutralizing antibody titers induced by CV7201 were evaluated, needle-free administration was superior to injection with a needle (57). In an influenza vaccine test, the intranodal (i.n.) delivery of naked mRNA elicited potent CD4 and CD8 T cell immune responses in mice, and repeated i.n. injection with modified mRNA led to priming antigen-specific CD4 and CD8 T cells, whereas subcutaneous, i.d. administration did not (96). Combination with two or more delivery methods have been explored and employed in cancer mRNA vaccine development. The combination of i.v. and i.d. injection of TriMix-DC-MEL therapy showed favorable outcomes in patients with broad CD8 and CD4 T cell immune responses (97). Further studies demonstrated that i.n. and intratumor injection with TriMix mRNA into dendritic cells achieved better therapeutic outcomes than alternate injection sites (98, 99). However, i.d. administration of RNActive vaccines presented a similar immune response to the i.n. administration of conventional mRNA vaccines, which was an inconsistent result (100). Altogether, these results highlight the importance of the delivery route for effective mRNA vaccines.

Similarly, Fleeton et al. showed that i.m. injection of *in vitro* transcribed naked self-amplifying mRNA based on the Semliki Forest virus genome could induce a protective immune response (62). Geall and colleagues showed that i.m. administration in mice and cotton rats with very low dose of self-amplifying mRNA encoding the F protein of respiratory syncytial virus (RSV) encapsulated with a synthetic LNP induced very high titers of IgG1 and interferon (IFN)-producing CD4 and CD8 T cells (22).

Delivery tools are equally important in the effectiveness of mRNA vaccines. Ideally, the delivery vehicle should protect RNA against potential digestion by ribonuclease and confer efficient target cell uptake, easy dissociation of RNA cargo from the vehicle and escape from the endosome. Overcoming the barrier of the cytoplasmic membrane and avoiding digestion by RNases are the initial steps for efficient RNA delivery into target cells. The final important requirements for an optimal delivery vehicle are a lack of both toxicity and immune stimulation. In initial studies, mRNA synthesized *in vitro* was directly injected into animals. Subsequently, mRNA vaccines formulated in liposomes were confirmed to induce a virus-specific anti-influenza cytotoxic T lymphocyte (CTL) immune response in mice (72). Several methods have been explored to increase delivery efficiency and great progress has been made in the field of designing delivery vehicles form RNA vaccines (101–103). In addition to the physical methods of gene guns and electroporation, mRNA vaccines have been delivered into the cytoplasm by cationic lipids and polymers. Cationic nano-emulsion formulated mRNA was also shown to induce a potent immune response (8, 23, 85). However, several of these delivery vehicles demonstrated toxicity *in vivo*, which may limit their use in humans (104). New platforms were developed as transportation tools for mRNA vaccines to avoid the limitation of toxic chemical transfection reagents. Most of these platforms utilized LNPs based on modified cationic lipid or lipid polymers. LNPs facilitate the delivery of RNA and enhances antigen expression dramatically. Several groups have utilized lipids or polymers as a platform to deliver mRNA vaccines against HIV-1 by a subcutaneous route, which efficiently elicited HIV-specific CD4 and CD8 T cell responses, or by an intranasal route, which induced an antigen-specific immune response (73, 105, 106). Lipid-encapsulated mRNA of influenza HA gene segments was also tested and showed T cell activation following a single dose (107). Combining LNP technology with nucleoside modification improves the efficacy of mRNA vaccines. LNP-formulated modified mRNA of influenza virus HA from H10N8 and H7N9 induced a potent protective immune response in mice, ferrets, and cynomolgus monkeys (108).

Another target against which LNP delivery of formulated mRNA has shown great potential is Zika virus. No vaccine is available to prevent this mosquito-borne disease and the recent epidemic has caused worldwide concern. Richner et al. reported that two vaccinations with LNP-encapsulated modified mRNA encoding a wild-type or edited prM-E gene induced a high order neutralizing antibody titer (75, 109).

Modified mRNA-based vaccines formulated with LNPs elicited robust immune responses and protected guinea pigs from Ebola virus disease as well (52). Intravenous (i.v.) injection with modified mRNA formulated in LNPs showed maximal protein expression at 6 h post-injection (110). Both i.d. and i.m. administration with non-replicating mRNA encoding influenza H10 encapsulated in LNPs induced high protective titers, but this response occurred more rapidly by i.d. administration than by i.m. administration (95).

LNPs are a popular delivery vehicle for self-amplifying mRNA vaccines well. A plethora of studies have shown that self-amplifying mRNA encapsulated in LNP induced potent cellular and humoral immune responses by different administration routes (19, 107, 111). LNP formulated self-amplifying mRNA vaccines encoding influenza virus antigens resulted in potent T and B cell immune responses and conferred protection against homologous and heterologous influenza virus challenges as well (63, 65, 112).

Cell penetrating peptides (CPPs), a type of cationic peptide, represent promising tools for mRNA delivery into intracellular target sites. Protamine is an arginine-rich cationic peptide that can bind to mRNA and transport it into cytoplasm. Protamine was extensively used as a delivery system for cancer and viral mRNA vaccines. The self-adjuvanted RNActive vaccine platform was created with protamine and has demonstrated potency against various infectious diseases and cancers (76, 77, 100). Recently, Coolen and colleagues designed innovative delivery platform consisting of poly(lactic acid) and cationic-penetrating peptides as mRNA condensing agent. This nano-complexes were taken up by dendritic cells induced strong protein expression and innate immune response (113).

Self-amplifying mRNA coding HA [A/California/ 07/2009(H1N1)] encapsulated into oil-in-water nano-emulsion stimulated protection against homologous and heterologous influenza virus (65). Formulation in polyethylenimine (PEI) of self-amplifying mRNA encoding H1N1/PR8-HA resulted in a significantly higher antibody titer and longer durable antigen expression than using non-formulated self-amplifying mRNA (31, 114). Chitosan and PEI were also utilized to deliver self-amplifying -mRNA as nanoparticles (114, 115). Chahal et al. developed an intriguing platform consisting of a chemically modified dendrimer nanoparticle to condense self-amplifying mRNA encoding influenza HA. A single immunization in mice elicited potent CD8+ T cell and antibody responses and protected mice against a broad spectrum of lethal pathogen challenges, including H1N1 influenza, Toxoplasma gondii, and Ebola virus (64).

Additional new modified nanoparticles are currently being investigated, such as polyplexes, nanoplexes and porous polymer scaffold-mediated delivery (116–122). Although great advances have been achieved in the development of delivery tools, the ideal platform maybe a combination of different mRNA delivery tools and more efforts in understanding mechanism of action might be required.

## Mechanism of Immune Response Induced by mRNA Vaccines

The immune response mechanism instigated by mRNA remains to be elucidated. The process of mRNA vaccine recognition by cellular sensors and the mechanism of sensor activation are still not clear. Intracellularly, two kinds of RNA sensors, endosomal toll-like receptors (TLRs) and the RIG-I-like receptor family, have been identified. The former set is divided into TLR-3, TLR7, TLR8, and TLR9, which are localized in the endosomal compartment of professional immune surveillance cells, such as DCs, macrophages and monocytes. TLR3 recognizes dsRNA longer than 45 base pair as well as dsRNA resulting from single strand RNA (ssRNA) forming secondary structures or derived from viral replication intermediates. TLR7 and TLR8 are activated by RNAs rich in polyuridines, guanosines and/or uridines. TLR7 can bind both dsRNA and single-stranded RNA (ssRNA), whereas TLR8 recognizes ssRNA only (123). TLR7 activation can increase antigen presentation, promote cytokine secretion and stimulate B cell responses (124). The latter family, functioning as a pattern recognition receptor (PRR), includes RIG-I, MDA5, and LGP2 (125). RIG-I preferentially recognize ssRNA and dsRNA bearing a 5′triphosphate, and stimulate IFN production (126–130). The panhandle structure in viral genome segments was directly involved IFNinduction through RIG-I activation (131). MDA5 is another cytosolic RNA sensor that detects long dsRNA generated during RNA virus replication (132) as well as RNA of synthetic origin, including poly I:C. Recognition by dsRNA induces the activation of IRF3 and NF-κB, subsequently leading to increased production of type I IFN (127, 133, 134). Sometimes, the elements of dsRNA recognized by PRR sensors can function as an adjuvant through the induction of IFN (135–137). mRNA vaccines can stimulate innate immunity through TLRs 3,7, and 8, RIG-I and MDA5 (138, 139). IFN induction by mRNA vaccines through RNA sensors is dependent on the quality of *in vitro* transcribed mRNA, delivery vehicle, and administration route. mRNA sensing by the innate immune system is a double-edged sword in the elimination of invading molecules. Natural exogenous mRNA stimulates strong induction of type I INFs and potent inflammatory cytokines, which instigate T and B immune responses but may negatively affect antigen expression (140–143). Interestingly, Blanchard and colleagues established a method to measure PRR activation by IVT mRNA in cells and in tissue section. In this method, proximity ligation assays (PLAs) was employed (144).

i.d. vaccination with the RNactive vaccine technology from CureVac AG, induced strong immune responses that are dependent on TLR7 signal. TLR7 activation leads to upregulation of chemokines, which in turn recruit innate immune cells such as DCs and macrophages to the site of injection (100). Activation of pro-inflammatory cytokines, such as TNF-α andIL-6 which are known to contribute to immune cells recruitment have been observed at the injection site (145).

On the other hand, an early shut-down of antigen expression after the mRNA vaccination due to PRRs activation might be detrimental. Consistently antigen expression, humoral and T cell responses to mRNA vaccination, both from conventional and amplifying mRNA, were significantly enhanced in IFNAR1/2 ^−/−^ mice (105, 146) or by co-administration of IFN antagonist (147). The negative impact from excessive IFN activation could derive not only from preventing RNA amplification, in case of self-amplifying mRNA vaccines, and expression, but also at the level of T cells. While type I IFN can determine the differentiation of antigen-primed CD8^+^ T cells into cytotoxic effectors, they may also promote T cell exhaustion (140). Whether type I IFN inhibits or stimulates the CD8 T cell response to mRNA vaccines might depend from the timing and intensity of type I IFN induced (140). T cell inhibition could prevail if triggering of type I IFN receptors precedes that of T cell receptors.

Modified mRNA with pseudouridines and mRNA purified with HPLC can reduce immune activation and increase antigen stability and expression (35, 148, 149). For an instance, i.p injection with mRNA containing pseudouridines induced antigen expression without the induction of cytokines in mice (150). Furthermore, some publications have shown that purity and delivery systems affect the immune response stimulated by mRNA vaccines (35). More interestingly, a recent study showed that modified mRNA encapsulated into LNPs has an adjuvant effect and induces a potent T follicular helper response and a large number of germinal center B cells with long-living, high affinity neutralizing antibodies (78).

Dentritic cell(DC) maturation is crucial to the effectiveness of mRNA-based vaccines. Generally, TLR7 was expresssed in plasmacytoid dentritic cells (pDCs) and B cells in humans, and TLR8 was expressed in conventional dentritic cells (cDCs), monocytes and macrophages. cDCs constitute the major resident DC population in normal human dermis and are characterized by CD1c expression (also known as blood dendritic cell antigen-1 (BDCA-1), whereas plasmacytoid DCs are present in the skin (129, 151, 152). The TLR7 and TLR8 locations in different DC subsets and DC locations in different organs may clarify the relationship between immune efficacy and the administration route and formulation of mRNA vaccines. Replacement of modified nucleotidesin mRNA decreased activation by binding mRNA to PRRs and reduced the innate immune response (153). mRNA vaccines not only stimulated the specific humoral immune response by the translated antigen but also the antigen-specific T cell response. Administration route and vaccine formulation determine the peak of antigen expression, which is another way to modulate the immune response (94, 154, 155). Liang et al. have shown that the kinetics of cell infiltration was largely similar between i.m. and i.d. administration in NHPs (156). The i.d. group showed stronger initial responses, probably because of rapid targeting, activation and transport to dLNs of skin DCs. Furthermore, only skin monocytes and DCs showed evidence of antigen translation at day 9, indicating prolonged antigen availability after i.d. delivery, and confirming the longer expression of mRNA-encoded antigen observed in mice (94).

A better elucidation of the sequence of events leading to mRNA translation and immune activation will help engineer mRNA vaccines to induce the correct balance of type I IFN induction, positively affecting vaccine outcome.

## Clinical Trials

Compared with the prophylactic and therapeutic application of mRNA in cancer, clinical trials of mRNA vaccines for infectious disease are still in their early age. Pilot clinical trials with DCs transfected with mRNA encoding various HIV-1 antigens, cellular molecules, or pp65 of human cytomegalovirus showed that mRNA vaccines are safe and that they elicited antigen-specific CD4^+^ and CD8^+^ T cell immune responses; however, no reduction of viral load was observed (157–160).

In a recent clinical trial of protamine complexed mRNA vaccine against rabies virus, the results showed that RNA complexed with protamine is safe and well-tolerated *in vivo*, but efficacy was highly dependent on the dose and route of administration. The efficacy of administration with a needle-free device was much better than with direct needle injection (57, 161). Results of a phase I showed LNP-formulated modified H10N8 mRNA vaccine induced robust humoral immune response in volunteers with mild or moderate adverse reaction (108).

## Prospective of RNA-based Vaccines

A plethora of publications have shown that mRNA-based vaccines are a promising novel platform that is high flexible, scalable, inexpensive, and cold-chain free. Most importantly, mRNA-based vaccines can fill the gap between emerging pandemic infectious disease and a bountiful supply of effective vaccines. A variety of preclinical and clinical projects have made enormous strides toward the conceivable application of mRNA vaccines and have suggested that mRNA-based prophylaxis and therapy can be translated to human applications. Although in medical application, magnitude of responses was lower than predicted from than those observed into animal models, the results of pilot clinical trials have shown good tolerability and that mRNA vaccination can induce antigen-specific T and B cell immune responses (57, 108). Therefore, mRNA holds great promises, but further insights into the mechanism of action and potency are still needed for full development of mRNA vaccines. The exploration of new strategies is needed to create applicable mRNA vaccines and to decrease the dose. As described above, the molecular impact of the innate immune response stimulated by mRNA through PAMP recognition is still not clear. Multiple efforts have been made to improve the stability and delivery efficiency of *in vivo* mRNA vaccine, including incorporation of 5′ and 3′ terminal untranslated regions and chemically modified nucleosides (162–164). Study demonstrated removal of dsRNA contaminants by high performance liquid chromatography purification of *in vitro* transcribed mRNA prolonged the translation (35). Research has demonstrated that modified nucleoside decreases the innate immune response and enhances protein expression. Optimization of the 5′-untranslated region (5′-UTR) of mRNA, whose secondary structures are recognized by cell-specific RNA binding proteins or PAMP molecules can maximize the translational yield of mRNA therapeutics and vaccines (43, 165). However, improper incorporation of modified nucleosides can have a negative impact on transcription products and increase costs.

Based on the results of the above described studies, a better understanding of the mechanism of action of mRNA vaccines, the identification and development of a new delivery system, and improvement of mRNA vaccine design will be attained (166).

mRNA vaccines have great potential and offer advantages over conventional vaccines. The growing body of preclinical and clinical results demonstrates that prophylaxis and therapy with mRNA promises to be useful for preventing infectious disease and treating tumors and that mRNA vaccines are safe and tolerated in animal models and humans. Additionally, future improvements should increase antigen-specific immune responses and the magnitude of memory immune cell responses, including memory B and T cell responses. Although mRNA vaccine technology has still not extensively tested in humans, publications of preclinical and early clinical tests have emerged in recent years, in which promising results were reported. This evoked the momentum of biocompanies to commercialize mRNA vaccines with great enthusiasm (167, 168). Some private funding resources and institutes have supported the research and development of mRNA vaccines (169, 170). Despite the need for further optimization of manufacturing processes to generate mRNA vaccines, these processes hopefully will be streamlined to be establish large-scale production. It is just a matter of time for RNA vaccines to be used in humans and animals.

## Author Contributions

CZ, JL, and HS wrote this manuscript. JL and GM revised this manuscript.

### Conflict of Interest Statement

GM is an employee of the GSK group of companies and reports ownership of GSK shares and/or restricted GSK shares. The remaining authors declare that the research was conducted in the absence of any commercial or financial relationships that could be construed as a potential conflict of interest.
